# Increased Intracranial Pressure Due to Transverse Sinus Compression by a Meningioma En Plaque

**DOI:** 10.7759/cureus.33487

**Published:** 2023-01-07

**Authors:** Aidan Healy, Upraj Singh, Sahibjot S Bhatia, Neuzil Lai, Forshing Lui

**Affiliations:** 1 Neurology, California Northstate University College of Medicine, Elk Grove, USA; 2 Neurology, Kaiser Permanente South Sacramento Medical Center, Sacramento, USA; 3 Clinical Sciences, California Northstate University College of Medicine, Elk Grove, USA

**Keywords:** venous sinus occlusion, meningioma en plaque, meningioma, lateral sinus thrombosis, idiopathic intracranial hypertension

## Abstract

Idiopathic intracranial hypertension (IIH) is defined as raised intracranial pressure of unknown etiology. Looking for underlying causes needs to be undertaken before the diagnosis is confirmed and managed accordingly. We are reporting a rare and unique case of a patient with the clinical diagnosis of idiopathic intracranial hypertension or pseudotumor cerebri. She is not obese and not taking any predisposing medication. Her first magnetic resonance imaging (MRI) and magnetic resonance venogram (MRV) were reported as showing left transverse sinus thrombosis. She was treated with anticoagulation. Her final diagnosis of left transverse sinus compression by a meningioma en plaque (MEP) was finally made with a repeat MRI. A high index of suspicion of unusual causes is important when IIH presents in an atypical patient or with an atypical presentation. The prognosis and management of real IIH and raised intracranial pressure due to other causes are different.

## Introduction

Idiopathic intracranial hypertension (IIH), which in the past was called pseudotumor cerebri, is defined as increased intracranial pressure (ICP) of unknown cause. All other causes of raised ICP need to be excluded before the diagnosis of IIH can be confirmed [1]. It is commonly associated with obesity, especially in women. The incidence of IIH in the general population is 1-3/100,000 in the general population and approximately 20/100,000 in obese women [2]. Other predisposing factors include pharmacological agents such as tetracyclines, hypervitaminosis A, and retinoids [3]. Venous sinus venography is usually performed to specifically look for underlying dural venous sinus thrombosis as the cause of raised ICP [4]. Meningioma is a slowly growing brain tumor. It may cause an increase in ICP due to its mass effect. Venous hypertension resulting in an increase in ICP is rare and only a few cases have been reported secondary to compression of the venous sinus [5]. We are reporting a rarer case of increased ICP due to external compression of the venous sinus by a rare type of meningioma, meningioma en plaque (MEP).

## Case presentation

**Figure 1:**
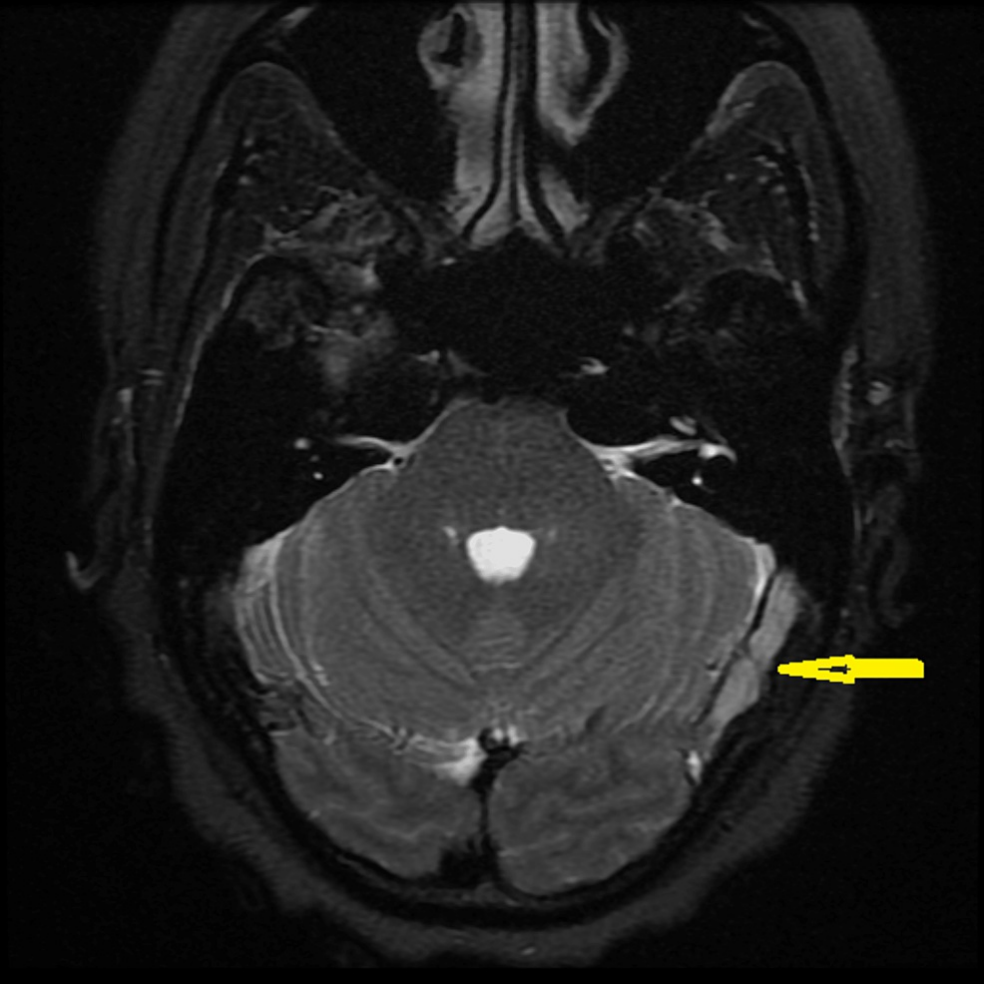
MRI head showing left transverse sinus thrombosis (yellow arrow)

The patient is a 42-year-old female with a known history of long-standing migraine without aura. She first presented to her primary care physician with a four-month history of a new kind of headache that was different from her previous migraines. Her new headache is mainly positional and occurred and worsened when lying down, coughing, sneezing, and exertion. She also complained of pulsatile tinnitus and intermittent blurring of vision with straining. She was not overweight, with a body mass index of 22. She was not taking any medication on a regular basis or recently, including tetracycline or vitamin A supplements. Because of her visual symptoms, she was evaluated by an ophthalmologist who found the presence of mild bilateral papilledema with normal visual acuity. The clinical diagnosis was idiopathic intracranial hypertension (IIH) or pseudotumor cerebri. Magnetic resonance imaging (MRI) of the head revealed left transverse sinus thrombosis (Figure 1).

**Figure 2:**
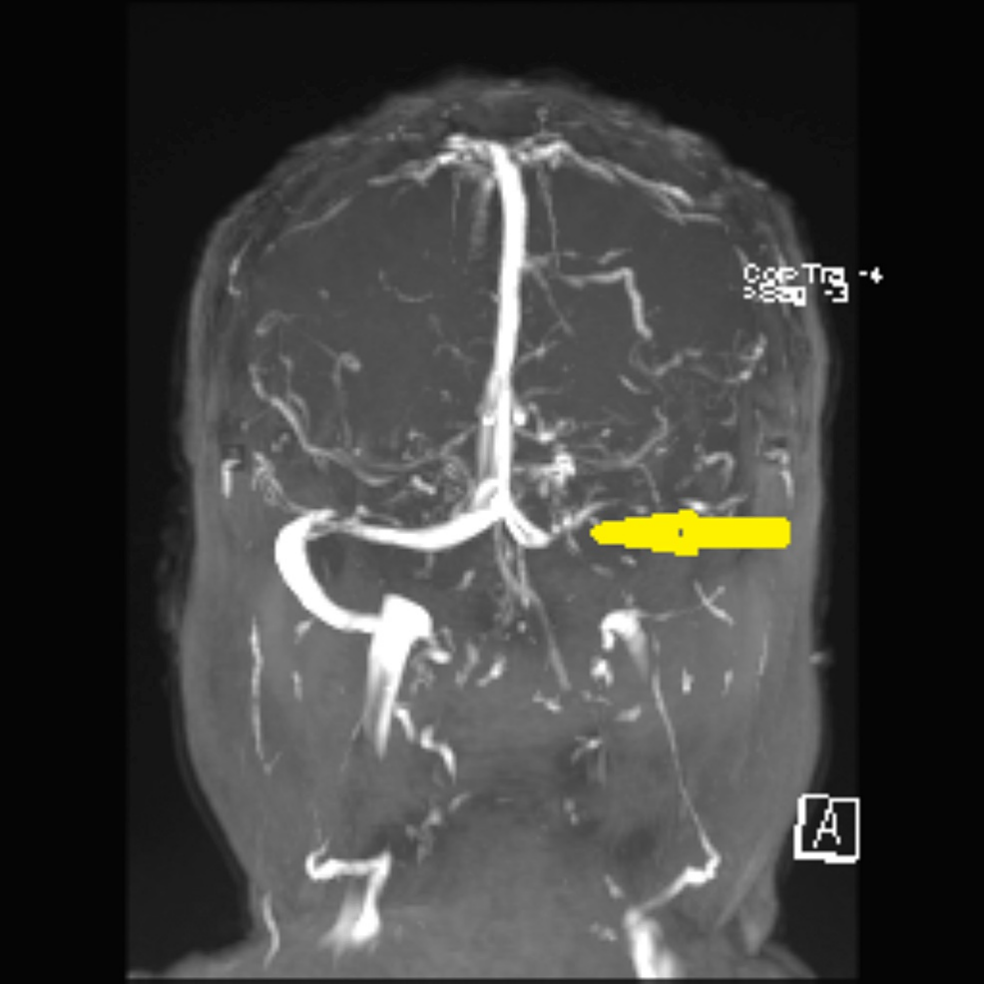
MR venogram showing left transverse sinus occlusion (yellow arrow)

Magnetic resonance venogram showed left transverse sinus occlusion (Figure 2).

She was started on treatment with dabigatran orally and referred to see a neurologist.

When she was seen in neurology, her symptoms and her exam findings were similar. Her medication list included dabigatran, varenicline, Flexeril, and verapamil. We agreed with the diagnosis of IIH-like clinical presentation secondary to left transverse sinus thrombosis. We recommended continuing with the dabigatran therapy and repeating the MRI head in three months.

**Figure 3:**
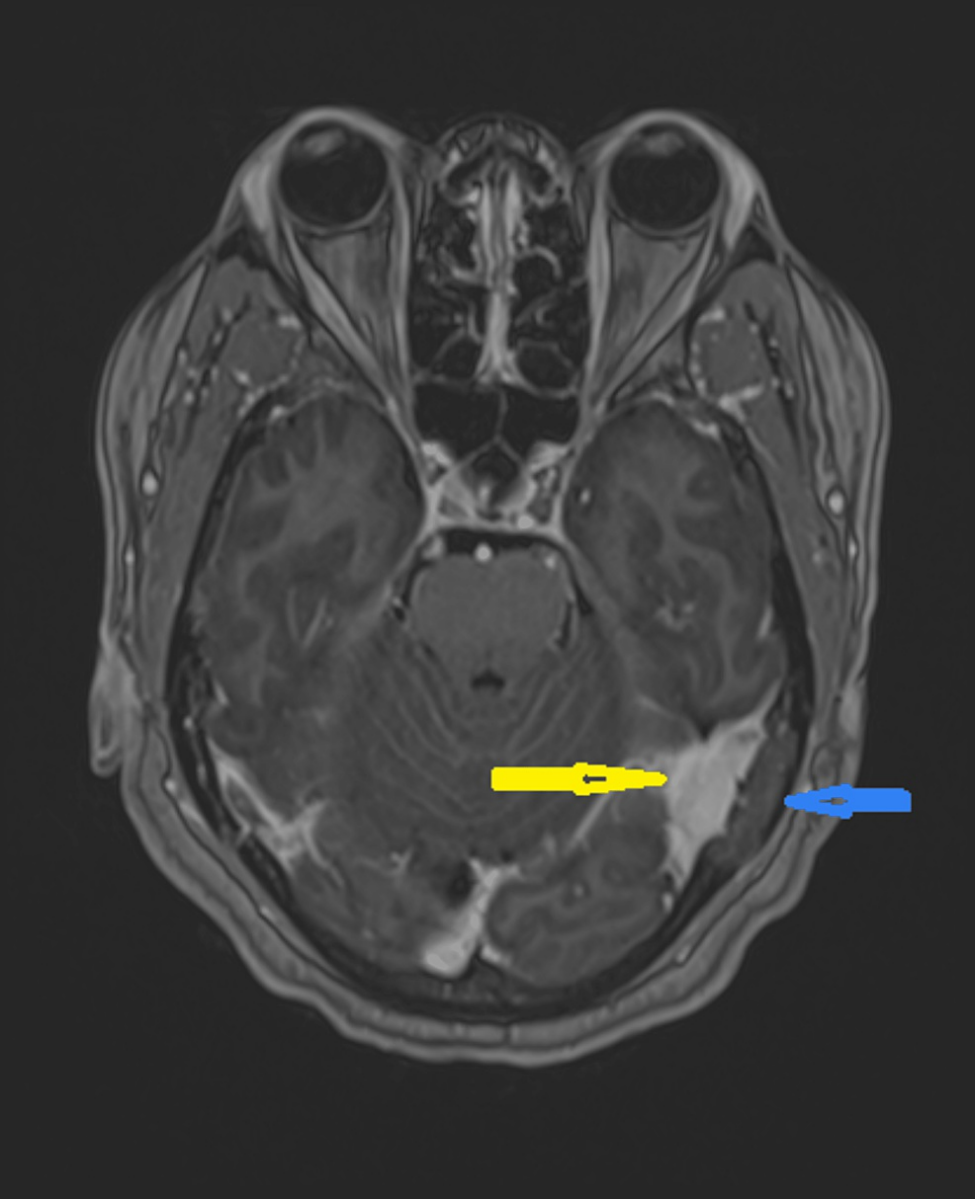
Follow-up MRI head with contrast enhancement Left temporal meningioma en plague (yellow arrow) and hyperostotic growth (blue arrow)

The follow-up MRI three months later unexpectedly showed an en plaque meningioma (MEP) with hyperostotic growths compressing a long segment of the transverse sinus (Figure 3).

The final diagnosis was occlusion of the left transverse sinus due to an underlying meningioma en plague and its accompanying hyperostosis. Her intracranial hypertension was clearly not idiopathic but secondary to left transverse sinus occlusion secondary to external compression.

## Discussion

Idiopathic intracranial hypertension (IIH), which, in the past, was called pseudotumor cerebri, is defined as increased intracranial pressure (ICP) of unknown cause. All other causes of raised ICP need to be excluded before the diagnosis of IIH can be confirmed [1]. The underlying pathogenesis of IIH is uncertain, with venous sinus stenosis as the most accepted cause because the commonest finding in venographic studies in patients with IIH is stenosis of the transverse sinus [3]. However, it is uncertain if venous sinus stenosis is the cause or consequence of increased intracranial pressure [6,7]. The recent discovery of glymphatics may provide an alternative to explain the altered CSF flow dynamics in IIH [6]. It is understandable that venous sinus occlusion or thrombosis will increase venous pressure, which is transmitted to the brain, resulting in increased ICP.

Based on her clinical features, with the exception of her normal BMI and lack of iatrogenic etiologies, her presentation was consistent with IIH. Our patient’s initial presentation is quite classic for IIH, with the exception that she is not obese and not on any medication associated with IIH. Her MRI head with MR venogram did show findings compatible with left transverse sinus thrombosis. Cerebral venous sinus thrombosis has different clinical presentations, and one type of presentation is like IIH. That is the reason why venographic studies are indicated in patients with a clinical diagnosis of IIH [4]. Our patient’s final cause of her lateral sinus occlusion turned out to be external compression of the lateral sinus by an MEP, which is very rare [5]. The final correct MRI interpretation was made with a follow-up imaging study.

Meningiomas are benign central nervous system tumors originating from the meninges' arachnoid cap cells. Clinical manifestations of the disease are determined by the location as well as the size of the mass. While most meningiomas are asymptomatic, symptomatic cases exhibit upper motor neuron signs as well as symptoms concomitant with increased intracranial pressure (anosmia, headaches, dizziness, visual impairments, seizures, papilledema, and behavioral changes) [8]. MEP refers to a meningioma that grows in a sheet-like manner, often involving overlying dura and bones with hyperostosis. These meningiomas constitute only 2% to 9% of all meningiomas, making them quite rare. MEPs are predominantly found in the spheno-orbital region and are associated with symptoms reflective of their location and degree of invasion [9,10]. Therefore, the most common symptoms of MEP in the sphenoid wing include ocular deficits, such as proptosis, followed by decreased visual acuity, retrobulbar pressure, visual field defects, headaches, orbital pain, temporal swelling, and periorbital swelling. In the temporal bone, hearing loss is the most prevalent symptom. However, lesser-known symptomatology, including dizziness, tinnitus, and otorrhea, have also been reported [11]. Our case displays a unique presentation of an already rare subclass of meningioma, MEP. This MEP was atypical in its location, as it compressed the transverse dural sinus with hyperostotic growth instead of an asymptomatic presentation in the sphenoid wings.

Our patient’s situation is unique in its rarity as well as its manifestation. Knowledge of the atypical manifestation may lead to a broader and more complete differential diagnosis when assessing subtle MEP or for clinical presentations of IIH. The knowledge may allow for a faster diagnosis and prompt management.

## Conclusions

In order to make a definitive diagnosis of idiopathic intracranial hypertension (IIH) or pseudotumor cerebri, other causes need to be considered and ruled out. The commonest known cause is dural sinus thrombosis, which requires specific treatment with anticoagulation on its own merit. Our case demonstrates a rare cause, i.e., transverse sinus occlusion due to external compression by an MEP, which is difficult to be diagnosed with neuroimaging studies. A high index of suspicion for unusual causes of sinus occlusion is necessary for earlier diagnosis and appropriate management and therapy. This often requires a good clinical neurologist and a competent neuroradiologist.
